# Risk of Hospitalization Associated with Cardiovascular Medications in the Elderly Italian Population: A Nationwide Multicenter Study in Emergency Departments

**DOI:** 10.3389/fphar.2020.611102

**Published:** 2021-01-29

**Authors:** Giada Crescioli, Alessandra Bettiol, Roberto Bonaiuti, Marco Tuccori, Marco Rossi, Annalisa Capuano, Silvia Pagani, Giulia Spada, Mauro Venegoni, Giuseppe Danilo Vighi, Guido Mannaioni, Alfredo Vannacci, Niccolò Lombardi, MEREAFaPS Study group

**Affiliations:** ^1^ Department of Neurosciences, Psychology, Drug Research and Child Health, Section of Pharmacology and Toxicology, University of Florence, Florence, Italy; ^2^ Tuscan Regional Center of Pharmacovigilance, Florence, Italy; ^3^ Department of Experimental and Clinical Medicine, University of Florence, Florence, Italy; ^4^ Joint Laboratory of Technological Solutions for Clinical Pharmacology, Pharmacovigilance and Bioinformatics, University of Florence, Florence, Italy; ^5^ Unit of Adverse Drug Reactions Monitoring, Department of Clinical and Experimental Medicine, University of Pisa, Pisa, Italy; ^6^ Campania Regional Center for Pharmacovigilance and Pharmacoepidemiology, Department of Experimental Medicine, Section of Pharmacology “L. Donatelli”, University of Campania “Luigi Vanvitelli,” Naples, Italy; ^7^ Internal Medicine, Medical Department, Vimercate Hospital, ASST Vimercate, Vimercate, Italy; ^8^ Hospital Pharmacy, Vimercate Hospital, ASST Vimercate, Vimercate, Italy; ^9^ Regional Center for Pharmacovigilance, Milan, Italy; ^10^ Toxicology Unit and Poison Center, Careggi University Hospital, Florence, Italy

**Keywords:** emergency department, hospitalization, adverse drug event, cardiovascular drug, elderly

## Abstract

**Background:** There is a significant gap in knowledge addressing cardiovascular (CV) medications safety in elderly. In this context, our purposes were to define clinical and pharmacological characteristics of outpatients’ adverse drug events (ADEs) related to CV medications leading to emergency department (ED) visits in the elderly Italian patients according to different age groups, and to evaluate the risk of hospitalization associated to ADEs in this population.

**Methods:** A multicentre, retrospective study was performed on reports of suspected ADEs collected between 2007–2018 in 94 EDs involved in the MEREAFaPS Study. Elderly patients who experienced one or more CV medications-related ADEs leading to ED visit were selected. Patients’ characteristics, suspected (ATC classes B and C) and concomitant drugs, and ADE description were collected. Elderly patients were stratified into three age groups (65–74, 75–84, and ≥85 years) and compared to adults (18–64 years). Logistic regression analyses were used to estimate the reporting odds ratios (RORs) with 95% confidence intervals (CIs) of ADE-related hospitalization adjusting for sex, presence of two or more suspected drugs, concomitant drugs, and one or more comorbidities.

**Results:** Among elderly, 16,926 reports of suspected ADE related to CV medications were collected, and 6,694 (39.5%) resulted in hospitalization. Patients were mostly female, Caucasians, and middle-old (75–84). 78.9% of patients were treated with only one suspected drug, and 71.9% and 47.1% reported concomitant medications and comorbidities, respectively. Compared to adults, risk of hospitalization was significantly higher for middle-old and oldest-old patients exposed to vitamin K antagonists (1.29 [1.09–1.52] and 1.56 [1.30–187]), direct thrombin inhibitors (3.41 [1.44–8.08] and 4.12 [1.67–10.17]), antiplatelets (1.51 [1.26–1.81] and 2.09 [1.71–2.57]), and beta-blockers (1.89 [1.38–2.59 and 2.31 [1.60–3.35]). Overall, a higher risk of hospitalization was observed for renin-angiotensin system inhibitors (1.32 [1.04–1.68], 1.65 [1.32–2.06], and 2.20 [1.70–2.85]), presence of two or more concomitant drugs, and concomitant conditions.

**Conclusion:** Our real-world findings underline relevant safety aspects of CV medications in the elderly Italian population. ED clinicians must always consider the higher risk of hospitalization related to the use of CV drugs in elderly, particularly in oldest-old ones, for antiarrhythmics, beta-blocking agents, renin-angiotensin system inhibitors, antiplatelets, and anticoagulants.

## Introduction

In the last century, the number of individuals aged 65 years and older increased significantly in high-income countries, as well as the number of patients affected by acute and chronic cardiovascular (CV) comorbidities (Christensen et al., 2009), many of which are characterized by a widespread use of CV medications for the prevention of morbidity and mortality (Fleg et al., 2011).

Elderly patients are known to be generally underrepresented in randomized clinical trials testing the efficacy and the safety of medications, including CV ones, and those who are usually included in the trials are often highly selected (Konrat et al., 2012), which limits generalization of the research findings to the general older populations.

Furthermore, as individuals age, elderly patients are at increased risk of developing adverse drug events (ADEs) (Perez et al., 2018) due to specific factors, including changes in drug metabolism as well as the presence of several concomitant medications, which can frequently lead to drug-drug and drug-disease interactions (Routledge et al., 2004; Davies and O’Mahony, 2015; Turgeon et al., 2017).

ADEs are an important cause of morbidity and emergency department (ED) visits and hospitalisations among the elderly (Budnitz et al., 2011). Although some studies have reported that the incidence of ADEs may be as high as 25% (Tecklenborg et al., 2020), a rate that is fourfold higher than in young adults (aged 18–64 years), the evidence that age is the sole predisposing factor for ADEs in the elderly is still debated (Davies and O’Mahony, 2015).

In evaluating real-world safety aspects of CV medications in the elderly, EDs can certainly represent a valuable observatory to perform pharmacovigilance active investigations about the clinical impact of ADEs in outpatients (Lombardi et al., 2018; Lombardi et al., 2020a; Lombardi et al., 2020b). Numerous investigations have been published on ED visits related to ADEs, but none of those found in the scientific literature have focused on CV medications in elderly.

Therefore, the purposes of the present study were to define the clinical and pharmacological characteristics of outpatients’ ADEs related to CV medications as cause of ED visits in the elderly Italian population, and to calculate the risk of hospitalization associated to ADEs in different elderly age groups compared to young adults.

## Materials and Methods

This is an observational retrospective study performed on data retrieved by pharmacovigilance reports of suspected ADE collected between January 1, 2007 and December 31, 2018 in the 94 EDs participating to the MEREAFaPS Study, an on-going multicentre study of active pharmacovigilance whose features have already been extensively described (Lombardi et al., 2020b). The involved hospitals belong to the territories of five Italian Regions: Lombardy and Piedmont (north), Tuscany and Emilia-Romagna (center), and Campania (south).

Within the MEREAFaPS Study database, all elderly patients (individuals aged 65 years and older) who experienced one or more CV medications-related ADEs leading to ED visit and hospitalization were selected and analyzed. Hospitalization was defined as an admission to the hospital following the ED visit. Independently from the time duration of ED stay, hospitalization was not considered when the patient was discharged after the visits.

For each elderly patient the following demographic, clinical, and pharmacological characteristics were evaluated: 1) age, gender, ethnicity; 2) clinical status on ED admission; 3) suspected and concomitant drugs (for each one, administration route, therapy duration, dosages, and therapeutic indication were recorded); 4) presence of concomitant conditions; 5) use of complementary and alternative medicines (CAM); 5) ADEs description; 6) ADEs outcome (in particular the presence or absence of ADE-related hospitalisation).

Anatomical Therapeutic Chemical (ATC) classification system was used to classify both suspected and concomitant drugs. ADEs reported from elderly outpatients having at least one clinical manifestation related to one or more CV medications were included in the analysis, considering only medications belonging to the ATC classes B and C, in particular: B01* (antithrombotic agents); B02* (antihemorrhagics); B03* (antianemic preparations); B05* (blood substitutes and perfusion solutions); C01* (cardiac therapy); C02* (antihypertensives); C03* (diuretics); C04* (peripheral vasodilators); C05* (vasoprotectives); C07* (beta blocking agents); C08* (calcium channel blockers); C09* (agents acting on the renin-angiotensin system); and C10* (lipid modifying agents). Patients who developed an ADE while in the ED for any other reason rather than CV medications were excluded. The Medical Dictionary for Regulatory Activities (MedDRA, version 21.0) was used to describe ADEs and comorbidities, that were coded and organized by System Organ Class (SOC) and Preferred Term (PT).

All cases extracted from the MEREAFaPS Study database were evaluated in order to assess the causality relationship between the suspected CV drugs and their related ADEs. Probability was assigned via a score termed definite (≥9), probable (5–8), possible (1–4) or doubtful (0) (Naranjo et al., 1981). This evaluation was performed by two groups of authors. In particular, GC and NL discussed each case independently from the evaluation performed by SP and GS. Any discrepancies were resolved by a third group of authors (MT, MR, AC, and AV). The application of the Naranjo score found a “possible” or “probable” association in most of the cases included in the present analysis.

Data were summarized using descriptive statistics. Categorical data were reported as frequencies and percentages and compared using the Chi-square test, while continuous data were reported as median values with the related interquartile ranges (IQRs) and compared using the Mann-Whitney test. Elderly patients were stratified according to the following age groups (Lee et al., 2018): group 1 (youngest-old), ranging from 65 to 74 years; group 2 (middle-old), ranging from 75 to 84 years; and group 3 (oldest-old), aged more than 85 years. For each CV medication group, as compared to all others belonging to the ATC classes of interest, univariate logistic regression was used to calculate the reporting odds ratios (RORs) of hospitalization with 95% confidence intervals (CIs) among each elderly group and compared to young adults (18–64 years). Multivariate logistic regression was performed and adjusted for sex, presence of two or more suspected drugs, presence of concomitant drugs, and presence of one or more comorbidities.

Adjustment was performed for all the above mentioned covariates. All results were considered to be statistically significant at *p* < 0.05. Data management and statistical analysis were carried out using STATA 16.

The coordinating center of Tuscany Region (Italy) approved the MEREAFaPS Study (Notification number 1225—December 21, 2009), and the local institutional ethics committee approved MEREAFaPS Study (Study number 3055/2010, Protocol number 45288—August 6, 2014) according to the legal requirements concerning observational studies. Due to the retrospective nature of the present study and data anonymization, patient’s consent to participate was not required.

## Results

Over the 12 years study period, a total of 61,855 ADE reports related to ED visits was assessed; of them, 16,926 (27.4%) were observed in elderly patients and related to CV medications (Youngest-Old n = 4,531; Middle-Old n = 8,006; Oldest-Old n = 4,389). Overall, 6,694 (39.5%) elderly patients were hospitalized due to the drug-related manifestation (Youngest-Old n = 1,463; Middle-Old n = 3,181; Oldest-Old n = 2050). Overall, we calculated that 40.1% (3,503/8,739) of female patients were hospitalized for ADEs related to CV medications vs 39.0% of male patients (3,191/8,187).

Table 1 reports demographic and clinical characteristics of elderly patients by age groups. Male patients were most represented in the youngest-old group (56.0%), while females were prevalent in middle-old (50.8%) and oldest-old (61.1%) groups. Overall, the majority of ADEs occurred in Caucasians and, at the time of adverse event, elderly patients were mostly treated with only one suspected drug. Among these, ATC class B was mostly reported in all elderly age groups (68.6, 73.1, and 73.1%), followed by medications belonging to the ATC class C (29.9, 25.3, and 25.0%). Concomitant drugs were reported in 67.7% of youngest-old, 72.4% middle-old, and 75.5% oldest-old. Most frequent concomitant drugs were those belonging to the cardiovascular system (ATC class C), followed by alimentary tract and metabolism (ATC class A), nervous system (ATC class N), blood and blood forming organs (ATC class B), and musculo-skeletal system (ATC class M). With increasing age, we observed an increase in the reported frequency for all ATC classes of concomitant drugs. The majority of patients among youngest-old (58.1%) and middle-old (52.6%) groups did not present concomitant conditions, while 52.5% of oldest patients reported to be affected by one or more comorbidities. Although with different percentages within the individual elderly age groups, the most frequently reported concomitant conditions were arterial hypertension, atrial fibrillation, ischaemic cardiomyopathy, dyslipidemia, and chronic renal failure. With increasing age, we observed an increase in the reported frequency for arterial hypertension, atrial fibrillation, and chronic renal failure. CAMs were reported in 1% of ADE reports. Moreover, with increasing age, we also observed an increase of the frequency of hospitalization among female patients. Among ED visits, a statistically significant difference was observed for all demographic and clinical characteristics analyzed, excluding the presence of CAMs. Demographic and clinical characteristics of young adults, who represent our comparison group, are described in Supplementary Table S1.

**TABLE 1:** Characteristics of elderly patients visiting the emergency department for an adverse drug event related to cardiovascular medications (ATC classes B and C).

Characteristics	Youngest-old	Middle-old	Oldest-old	*p*-value
	65–74 years	75–84 years	≥85 years	
	N = 4,531 (%)	N = 8,006 (%)	N = 4,389 (%)	
Sex				
Female	1,993 (44.0)	4,065 (50.8)	2,681 (61.1)	<0.001
Male	2,538 (56.0)	3,941 (49.2)	1,708 (38.9)	
Patients’ ethnicity				
Asian	16 (0.4)	9 (0.1)	2 (0.1)	<0.001
Black or African-American	6 (0.1)	5 (0.1)	0	
Caucasian	4,115 (90.8)	723 (90.4)	3,900 (88.9)	
Others	7 (0.2)	8 (0.1)	3 (0.1)	
Not available	387 (8.5)	750 (9.4)	484 (11.0)	
No. of suspected drugs involved in ADE				
1	3,494 (77.1)	6,388 (79.8)	3,488 (79.5)	0.006
2	782 (17.3)	1,230 (15.4)	669 (15.2)	
>3	255 (5.6)	388 (4.9)	232 (5.3)	
ATC class of suspected drugs				
ATC class B	3,107 (68.6)	5,855 (73.1)	3,208 (73.1)	<0.001
ATC class C	1,354 (29.9)	2,026 (25.3)	1,095 (25.0)	
Both classes	70 (1.5)	125 (1.6)	86 (2.0)	
Concomitant drugs				
No	1,464 (32.3)	2,207 (27.6)	1,074 (24.5)	<0.001
Yes	3,067 (67.7)	5,799 (72.4)	3,315 (75.5)	
No. of concomitant drugs				
0	1,464 (32.3)	2,207 (27.6)	1,074 (24.5)	<0.001
1	595 (13.1)	845 (10.6)	386 (8.8)	
2	503 (11.1)	818 (10.2)	445 (10.1)	
3–4	867 (19.1)	1,607 (20.1)	1,031 (23.5)	
>5	1,102 (24.3)	2,529 (31.6)	1,453 (33.1)	
ATC class of most frequently reported concomitant drugs				
ATC class C	2,407 (53.1)	4,785 (59.8)	2,808 (64.0)	
ATC class A	1,450 (32.0)	2,915 (36.4)	1,723 (39.3)	
ATC class N	743 (16.4)	1,817 (22.7)	1,183 (27.0)	
ATC class B	816 (18.0)	1,700 (21.2)	996 (22.7)	
ATC class M	386 (8.5)	936 (11.7)	592 (13.5)	
Concomitant conditions				
No	2,632 (58.1)	4,210 (52.6)	2,086 (47.5)	<0.001
Yes	1,899 (41.9)	3,796 (47.4)	2,303 (52.5)	
No. of concomitant conditions				
0	2,632 (58.1)	4,210 (52.6)	2,086 (47.5)	<0.001
1	756 (16.7)	1,306 (16.3)	783 (17.8)	
2	454 (10.0)	838 (10.5)	493 (11.2)	
>3	689 (15.2)	1,652 (20.6)	1,027 (23.4)	
Most frequently reported concomitant conditions^b^				
Arterial hypertension	850 (18.8)	1,698 (21.2)	969 (22.1)	
Atrial fibrillation	316 (7.0)	905 (11.3)	611 (13.9)	
Diabetes	296 (6.5)	617 (7.7)	280 (6.4)	
Ischaemic cardiomyopathy	255 (5.6)	470 (5.9)	238 (5.4)	
Dyslipidaemia	207 (4.6)	376 (4.7)	136 (3.1)	
Chronic renal failure	169 (3.7)	470 (5.9) ↑	370 (8.4) ↑	
COPD	87 (1.9)	252 (3.1)	135 (3.1)	
Presence of CAM				
No	4,483 (98.9)	7,940 (99.2)	4,343 (99.0)	0.305
Yes	48 (1.1)	66 (0.8)	46 (1.1)	
Hospitalization				
No	3,068 (67.7)	4,825 (60.3)	2,339 (53.3)	<0.001
Female	1,416 (46.1)	2,417 (50.1)	1,403 (60.0)	
Male	1,652 (53.9)	2,408 (49.9)	936 (40.0)	
Yes	1,463 (32.3)	3,181 (39.7)	2,050 (46.7)	<0.001
Female	577 (39.4)	1,648 (51.8)	1,278 (62.3)	
Male	886 (60.6)	1,533 (48.2)	772 (37.7)	

ADE, adverse drug event; ATC, anatomical therapeutic chemical; CAM, complementary and alternative medicine; COPD, chronic obstructive pulmonary disease.

^a^ Most frequently reported concomitant drugs (as ATC class, first level): A, alimentary tract and metabolism; B, blood and blood forming organs; C, cardiovascular system; M, musculo-skeletal system; N, nervous system.

^b^ Most frequently reported concomitant conditions (as preferred terms) out of 20,824 reported low-level terms MedDRA.

**FIGURE 1:**
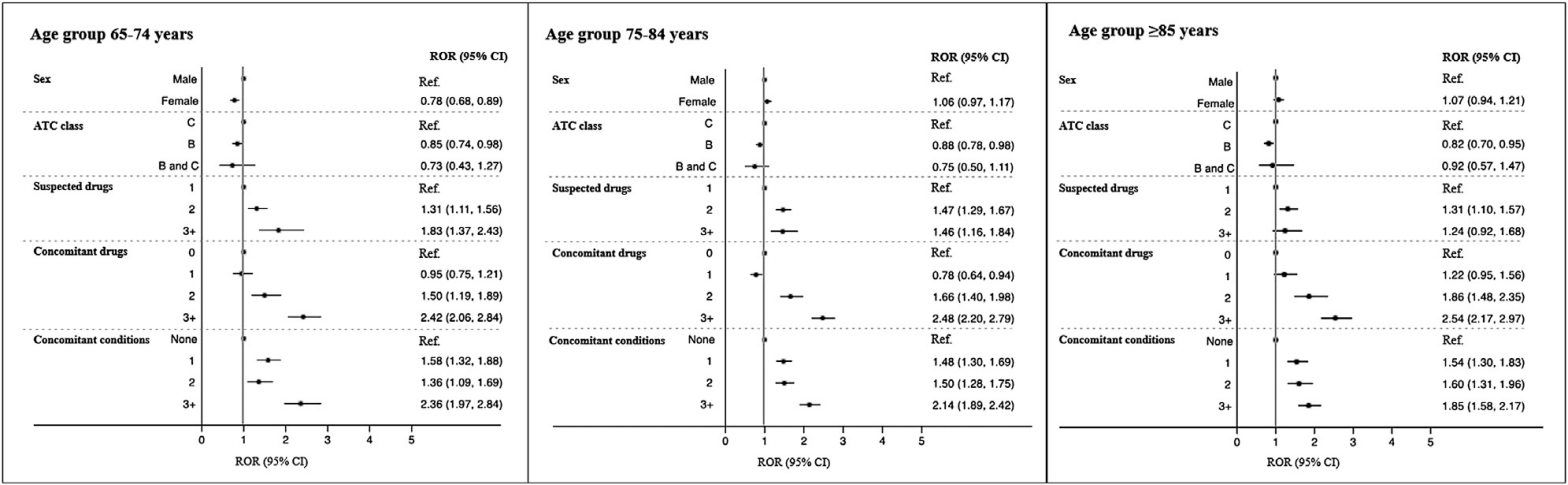
Predictors of hospitalization among the elderly age groups expressed as reporting odds ratio (ROR). CAM, complementary and alternative medicine.

Table 2 reports the most frequently reported CV medication groups and risk of hospitalization for elderly patients by age groups. Out of the total of suspected drugs, 68.3% belonged to the ATC class B and 31.7% to the ATC class C. In particular, anticoagulants, antiplatelets, and renin-angiotensin system inhibitors were the pharmacological groups most represented among the three study cohorts. Among ATC class B, the risk of hospitalization was significantly higher for middle-old and oldest-old patients compared to young adults for vitamin K antagonists (ROR 1.29, 95% CI [1.09–1.52] and 1.56 [1.30–1.87]), direct thrombin inhibitors (3.41 [1.44–8.08] and 4.12 [1.67–10.17]), acetylsalicylic acid (1.45 [1.19–1.77] and 1.99 [1.59–2.48]), and platelet P2Y_12_ receptor antagonists (1.57 [1.14–2.17] and 2.37 [1.63–3.44]). Considering ATC class B, the risk of hospitalization was significantly higher for all elderly age groups compared to young adults for renin-angiotensin system inhibitors (1.32 [1.04–1.68], 1.65 [1.32–2.06], and 2.20 [1.70–2.85]). Middle-old and oldest-old patients were at higher risk of hospitalization if exposed to beta blocking agents (1.89 [1.38–2.59] and 2.31 [1.60–3.35]), while only oldest-old patients were at higher risk if exposed to diuretics (1.54 [1.07–2.22]) and to antiarrhythmics (2.80 [1.42–5.54]). Furthermore, adjusted multivariate logistic regression indicated that the risk of hospitalization was significantly higher for all elderly age groups compared to young adults as the number of suspected and concomitant drugs, and the number of concomitant conditions increases (Figure 1).

**TABLE 2:** Suspected cardiovascular medication groups (ATC classes B and C) and risk of hospitalization for elderly patients.

Cardiovascular medication groups	Youngest-old	Middle-old	Oldest-old	Youngest-old	Middle-old	Oldest-old
	65–74 years	75–84 years	≥85 years	65–74 years	75–84 years	>85 years
	N = 4,531 (%)	N = 8,006 (%)	N = 4,389 (%)	ROR (95%CI)^a^	ROR (95%CI)^a^	ROR (95%CI)^a^
ATC class B, blood and blood forming organs						
Anticoagulants	2,024 (44.7)	4,119 (51.5)	2,210 (50.4)	1.02 (0.86–1.20)	1.35 (1.16–1.56)	1.61 (1.37–1.89)
Vitamin K antagonists (warfarin)	1,756 (38.8)	3,473 (43.4)	1,833 (41.8)	0.97 (0.81–1.17)	1.29 (1.09–1.52)	1.56 (1.30–1.87)
Factor Xa inhibitors	75 (17.7)	231 (2.9)	145 (3.3)	1.28 (0.46–3.54)	1.33 (0.52–3.42)	1.35 (0.51–3.58)
Unfractionated and low-molecular-weight heparins	143 (3.2)	251 (3.1)	142 (3.2)	1.15 (0.71–1.87)	1.49 (0.97–2.30)	1.63 (1.00–2.65)
Direct thrombin inhibitors	83 (1.8)	218 (2.7)	111 (2.5)	2.14 (0.83–5.49)	3.41 (1.44–8.08)	4.12 (1.67–10.17)
Antiplatelets	1,189 (26.2)	1,905 (23.8)	1,074 (24.5)	1.17 (0.95–1.42)	1.51 (1.26–1.81)	2.09 (1.71–2.57)
Acetylsalicylic acid	984 (21.7)	1,513 (18.9)	852 (19.4)	1.15 (0.92–1.43)	1.45 (1.19–1.77)	1.99 (1.59–2.48)
Platelet P2Y_12_ receptor antagonists	375 (8.3)	560 (7.0)	273 (6.2)	1.00 (0.71–1.42)	1.57 (1.14–2.17)	2.37 (1.63–3.44)
Enzymes	3 (0.1)	4 (0.1)	—	—	—	—
Other blood agents^b^	22 (0.5)	41 (0.5)	27 (0.6)	0.43 (0.12–1.53)	1.16 (0.50–2.71)	1.74 (0.67–4.54)
ATC class C, cardiovascular system						
Renin-angiotensin system inhibitors	620 (13.7)	893 (11.2)	469 (10.7)	1.32 (1.04–1.68)	1.65 (1.32–2.06)	2.20 (1.70–2.85)
Diuretics	221 (4.9)	509 (6.4)	425 (9.7)	1.13 (0.76–1.70)	1.21 (0.85–1.72)	1.54 (1.07–2.22)
Beta blocking agents	327 (7.2)	467 (5.8)	227 (5.2)	1.16 (0.82–1.63)	1.89 (1.38–2.59)	2.31 (1.60–3.35)
Calcium channel blockers	190 (4.2)	262 (3.3)	99 (2.3)	1.15 (0.72–1.85)	1.42 (0.91–2.19)	1.09 (0.61–1.95)
Antiarrhythmics	118 (2.6)	190 (2.4)	73 (1.7)	1.46 (0.80–2.64)	1.63 (0.94–2.83)	2.80 (1.42–5.54)
Lipid modifying agents	84 (1.9)	73 (0.9)	19 (0.4)	1.32 (0.74–2.34)	1.44 (0.79–2.63)	2.02 (0.73–5.61)
Digitalis glycosides	28 (0.6)	124 (1.6)	154 (3.5)	0.66 (0.13–3.26)	1.62 (0.38–6.94)	1.03 (0.24–4.40)
Antiadrenergic agents	81 (1.8)	94 (1.2)	30 (0.7)	0.88 (0.43–1.82)	0.72 (0.35–1.49)	1.22 (0.48–3.15)
Other cardiovascular agents^c^	220 (4.9)	346 (4.3)	146 (3.3)	2.10 (1.42–3.09)	2.46 (1.73–3.52)	2.68 (1.72–4.17)

ATC, anatomical therapeutic chemical; ROR, reporting odds ratio.

^a^ As compared to adults (18–64 years); models are adjusted for sex, presence of 2+ suspected drugs, presence of concomitant drugs, and presence of 1+ comorbidities.

^b^ Other blood agents: B02*; B03*; B05*.

^c^ Other cardiovascular agents: C01* (excl. C01AA*); C04*; C05*.

Table 3 reports the most frequently reported suspected drugs among CV medications leading to ED visit. The total number of suspected drugs analyzed was 20,070, of which 13,700 (3,586 youngest-old; 6,587 middle-old; 3,527 oldest-old) belonging to the ATC class B and 6,370 (1829 youngest-old; 2,885 middle-old; 1,656 oldest-old) to the ATC class C. Although with different percentages within the individual elderly age groups, ATC class B was mostly represented by warfarin (39.7%), acetylsalicylic acid (24.4%), clopidogrel (5.4%), acenocumarol (4.5%), and dabigatran (3.0%). Similarly, in terms of reported frequencies for each elderly age group, ATC class C was mostly represented by furosemide (9.7%), ramipril (7.7%), bisoprolol (5.1%), digoxin (4.2%), and amiodarone (4.1%).

**TABLE 3:** Suspected drugs among cardiovascular medications (ATC classes B and C) leading to emergency department visit.

Suspected drugs	Elderly overall	Youngest-old	Middle-old	Oldest-old
		65–74 years	75–84 years	>85 years
ATC class B, blood and blood forming organs	N = 13,700 (%)	N = 3,586 (%)	N = 6,587 (%)	N = 3,527 (%)
Warfarin	5,445 (39.7)	1,614 (45.0)	2,178 (48.3)	1,653 (46.9)
Acetylsalicylic acid	3,349 (24.4)	984 (27.4)	1,513 (23.0)	852 (24.2)
Clopidogrel	737 (5.4)	242 (6.8)	333 (5.1)	162 (4.6)
Acenocoumarol	617 (4.5)	142 (4.0)	295 (4.5)	180 (5.1)
Dabigatran	412 (3.0)	83 (2.3)	218 (3.3)	111 (3.2)
Rivaroxaban	387 (2.8)	83 (2.3)	207 (3.1)	97 (2.8)
Enoxaparin	383 (2.8)	100 (2.8)	179 (2.7)	104 (3.0)
Ticlopidine	356 (2.6)	69 (1.9)	184 (2.8)	103 (2.9)
Apixaban	184 (1.3)	27 (0.8)	83 (1.3)	74 (2.1)
Edoxaban	91 (0.7)	—	53 (0.8)	38 (1.1)
Ticagrelor	50 (0.4)	50 (1.4)	—	—
ATC class C, cardiovascular system	N = 6,370 (%)	N = 1,829 (%)	N = 2,885 (%)	N = 1,656 (%)
Furosemide	616 (9.7)	98 (5.4)	274 (9.5)	244 (14.7)
Ramipril	488 (7.7)	145 (7.9)	221 (7.66)	122 (7.4)
Bisoprolol	327 (5.1)	85 (4.7)	134 (4.6)	108 (6.5)
Digoxin	270 (4.2)	—	121 (4.2)	149 (9.0)
Amiodarone	259 (4.1)	65 (3.6)	139 (4.8)	55 (3.3)
Amlodipine	223 (3.5)	96 (5.3)	127 (4.4)	—
Enalapril	213 (3.3)	57 (3.1)	101 (3.5)	55 (3.3)
Doxazosin	151 (2.4)	71 (3.9)	80 (2.8)	—
Atenolol	139 (2.2)	65 (3.6)	74 (2.6)	—
Metoprolol	110 (1.7)	44 (2.4)	66 (2.3)	—
Hydrochlorothiazide	61 (1.0)	—	—	61 (3.7)
Spironolactone	47 (0.7)	—	—	47 (2.8)
Canrenone	46 (0.7)	—	—	46 (2.8)
Valsartan and diuretic	46 (0.7)	—	—	46 (2.8)
Carvedilol	43 (0.7)	43 (2.4)	—	—

ATC, anatomical therapeutic chemical.

Table 4 reports ADEs associated with the most frequently reported suspected CV medication groups leading to ED visit. The total number of PT analyzed was 27,497, of which 18,251 (4,517 youngest-old; 8,710 middle-old; 5,024 oldest-old) belonging to the ATC class B and 9,246 (2,733 youngest-old; 4,151 middle-old; 2,362 oldest-old) to the ATC class C. Although with different percentages within the individual elderly age groups, ATC class B was mostly associated to epistaxis (17.0%), gastrointestinal bleedings (13.2%), alterations of the international normalized ratio (8.1%), central nervous system hemorrhages (6.1%), and genitourinary bleedings (5.7). Similarly, ATC class C was mostly associated to hypotension, syncope and pre-syncope (16.7%), electrolyte imbalance (13.2%), bradycardia (6.4%), asthenia and muscular weakness (5.2%), and dermatologic reactions (4.4%).

**TABLE 4:** Adverse drug events among cardiovascular medications (ATC classes B and C) leading to emergency department visit.

Adverse drug event	Elderly overall	Youngest-old	Middle-old	Oldest-old
		65–74 years	75–84 years	>85 years
ATC class B, blood and blood forming organs	N = 18,251 (%)	N = 4,517 (%)	N = 8,710 (%)	N = 5,024 (%)
Haemorrhage	9,131 (50.0)	2,450 (54.2)	4,243 (48.7)	2,438 (48.5)
Epistaxis	3,108 (17.0)	905 (20.0)	1,436 (16.5)	767 (15.3)
Gastrointestinal	2,414 (13.2)	683 (15.1)	1,026 (11.8)	705 (14.0)
Central nervous system	1,106 (6.1)	202 (4.5)	605 (6.9)	299 (6.0)
Genitourinary	1,043 (5.7)	254 (5.6)	499 (5.7)	290 (5.8)
Dermatologic	743 (4.1)	186 (4.1)	335 (3.8)	222 (4.4)
Ophthalmic	330 (1.8)	102 (2.3)	148 (1.7)	80 (1.6)
Pulmonary	191 (1.0)	52 (1.1)	107 (1.2)	32 (0.6)
Not specified	196 (1.1)	66 (1.5)	87 (1.0)	43 (0.9)
Altered international normalized ratio	1,482 (8.1)	283 (6.3)	635 (7.3)	564 (11.2)
Anaemia	1,011 (5.5)	199 (4.4)	446 (5.1)	366 (7.3)
Unintentional or intentional overdose	342 (1.9)	79 (1.7)	138 (1.6)	125 (2.5)
ATC class C, cardiovascular system	N = 9,246 (%)	N = 2,733 (%)	N = 4,151 (%)	N = 2,362 (%)
Hypotension, syncope and pre-syncope	1,541 (16.7)	430 (15.7)	748 (18.0)	363 (15.4)
Electrolyte imbalance	1,219 (13.2)	265 (9.7)	561 (13.5)	393 (16.6)
Hyponatremia	529 (5.7)	109 (4.0)	248 (6.0)	172 (7.3)
Hyperkalaemia	365 (3.9)	66 (2.4)	163 (3.9)	136 (5.8)
Hypokalaemia	325 (3.5)	90 (3.3)	150 (3.6)	85 (3.6)
Bradycardia	590 (6.4)	144 (5.3)	276 (6.6)	170 (7.2)
Asthenia and muscular weakness	477 (5.2)	137 (5.0)	216 (5.2)	124 (5.2)
Dermatologic reaction	403 (4.4)	164 (6.0)	169 (4.1)	70 (3.0)
Erythema	201 (2.2)	67 (2.5)	91 (2.2)	43 (1.8)
Localized or general pruritus	106 (1.1)	45 (1.6)	47 (1.1)	14 (0.6)
Urticaria	96 (1.0)	52 (1.9)	31 (0.7)	13 (0.6)
Localized or peripheral edema	399 (4.3)	161 (5.9)	175 (4.2)	63 (2.7)
Dizziness	252 (2.7)	85 (3.1)	120 (2.9)	47 (2.0)

ATC, anatomical therapeutic chemical.

## Discussion

This active pharmacovigilance study was carried out to define the clinical and pharmacological characteristics of outpatients’ ADEs associated with CV medications leading to ED visits in the elderly Italian population. To our knowledge, this is the first analysis of its kind conducted in several Italian EDs to calculate the risk of hospitalization related to CV medications in different elderly age groups compared to young adults.

From an in-depth literature search, numerous investigations have been reported on ADEs leading to ED visits and hospitalizations in European high-income countries (Lombardi et al., 2020b). A French survey (Queneau et al., 2007), performed over two periods of 1 week each, in EDs of five university hospitals and five general hospitals throughout France, reported that 21% of patients needed a clinical consultation after experiencing an ADE. The authors included all patients aged ≥15 years, without performing age subgroup analyses. Noteworthy, their multivariate logistic regression analysis found that age and number of concomitant medications were significantly associated with the ADE. In particular, the most frequently incriminated drug classes included diuretics (11.7%), anticoagulants (9.3%) and other CV drugs (15.4%).

Another prospective study performed in France (four non-consecutive weeks in 2002–2003) aimed to assess the incidence of adverse drug reactions (ADRs) and to identify the factors associated to hospital admissions in the elderly population (Olivier et al., 2009). Authors compared the characteristics of patients admitted for a suspected ADR with those of patients admitted for other reasons. They found that the number of drugs being taken (OR 1.18, 95% CI [1.08, 1.29]) and the use of antithrombotic agents (2.26, [1.33, 3.88]) were the factors most frequently related to ADRs.

Rodenburg and colleagues conducted a nationwide study of all hospital admissions between 2000 and 2005 with data from the Dutch National Medical Register with the aim of studying the differences between men and women in hospital admissions for ADRs due to CV drugs (Rodenburg et al., 2012). Overall, 34% of all ADR-related admissions were attributed to CV drugs, with a prevalence of female sex (54%). Similarly, to our study, the authors found that anticoagulants and antiplatelets, particularly salicylates, diuretics, and cardiotonic glycosides were responsible for the majority of the ADR-related hospital admissions.

A small prospective cross-sectional diagnostic study (30-days follow-up) was performed in the ED of the University Hospital of Basel (Switzerland) to identify the frequency of drug-related problems (DRPs) among elderly patients presenting to the ED with non-specific complaints (NSC), and to evaluate responsible drug classes (Nickel et al., 2013). During the study period, 633 NSC patients were included. Their median age was 81 years (IQR 72–87), and authors reported a mean Charlson comorbidity index of 2.5 (IQR 1–4). DRPs were identified in 12.2% of cases. Polypharmacy and diuretics, in particular thiazides, were most frequently associated with DRPs.

In four large German hospitals, the percentage of suspected ADR cases among all adult patients presenting to the ED was determined during a 30 days period study (Schurig et al., 2018). The authors analyzed a total of 10,174 emergency room visits, 665 of which were potentially associated with a suspected ADR. The median age of the study population was 74.5 years, and 264 patients (75%) were 65 years old or older, and 55% were women. Patients with ADR were found to be taking a median of seven different drugs simultaneously and, similarly to our study, antithrombotic agents, beta-blockers, renin-angiotensin system inhibitors, and diuretics were the most commonly suspected cause of ADR.

Through a review of observational studies, Bouvy and colleagues underlined that the occurrence of ADEs within the European hospital setting is still significant (Bouvy et al., 2015). However, the still low number of studies performed in outpatients, such as the investigations on ADEs leading to ED visits and hospitalisations, particularly those performed on a large sample, identify a scarcity of information on ADEs epidemiology in this setting.

In Italy, in a retrospective cohort study of data from an active pharmacovigilance project at 32 EDs in the Lombardy region collected between January 1, 2010 and December 31, 2011, Perrone and co-workers assessed the preventability, seriousness, and economic burden of ADRs as cause of ED admission (Perrone et al., 2014). During the study period, the authors analyzed 8,862 ADRs and found that B (blood and blood-forming organs) was one of the most frequently reported ATC class leading to ED admissions. Furthermore, older age and polypharmacy were associated with a higher risk of hospitalization. These findings have already been confirmed in our large nationwide multicentre study published in 2020 (Lombardi et al., 2020b).

Comparing our results with those obtained from the American and Asian high-income countries, it seems quite clear that important differences exist both in terms of study methods and study population. Nevertheless, most of the evidence published from these studies on the safety of CV medications in the elderly visiting the ED are quite comparable to those reported in our analysis, in particular in terms of suspected drug classes and other risk factors (i.e., high number of concomitant drugs and/or concomitant conditions).

A cross sectional study, aimed to address the association between inappropriate prescribing in elderly Medicare/Medicaid dual enrolees and injury-related ED visit (Blackwell et al., 2009), found that CV agents had the lowest proportion of ED-related fills for injuries compared to the other drug categories. However, among CV agents, clonidine and doxazosin had higher associations with injury than nifedipine. Additionally, based on cost, doxazosin was associated with the most expensive injury-related ED visits in the category of CV medications.

Between 2004–2005, a nationally representative, public health surveillance of ADEs and a cross-sectional survey of outpatient medical visits were performed to estimate the number of and risk for ED visits for ADEs involving Beers criteria medications compared with other medications (Budnitz et al., 2007). Among elderly U.S. patients, an estimated 177,504 ED visits for ADEs occurred both years. An estimated 3.6% of these visits were caused by adverse events related to medications considered to be always potentially inappropriate, according to the Beers criteria, and 33.3% of visits were for adverse events from three other medications, including warfarin (17.3%) and digoxin (3.2%). The authors also concluded that performance measurements and interventions targeting warfarin and digoxin use could prevent multiple ED visits for ADE.

Budnitz and colleagues performed another nationally representative study, using the adverse-event data from the National Electronic Injury Surveillance System-Cooperative Adverse Drug Event Surveillance project to estimate the frequency and rates of hospitalization after ED visits for ADEs in older Americans and to assess the contribution of specific medications (Budnitz et al., 2011). On the basis of 5,077 cases identified in their sample, there were an estimated 99,628 emergency hospitalizations for ADEs each year from 2007 through 2009. Nearly half of these hospitalizations were among middle-old and oldest-old patients (48.1%). Medications or medication classes most frequently implicated alone or in combination in 67.0% of hospitalizations were warfarin (33.3%) and oral antiplatelet agents (13.3%). Budnitz reported that the majority of emergency hospitalizations for recognized ADEs in older Americans resulted from a few commonly used medications, concluding that better management of antithrombotic therapies could have the potential to reduce ADE-related hospitalizations in the elderly.

In a relevant publication, Budnitz and colleagues also described the characteristics of ED visits for ADEs in the United States in 2013–2014, performing an active, nationally representative, public health surveillance in 58 EDs participating in the National Electronic Injury Surveillance System-Cooperative Adverse Drug Event Surveillance project (Shehab et al., 2016). Based on data from 42,585 cases, an estimated 34.5% of ED visits for ADEs occurred among adults aged 65 years or older in 2013–2014 compared with an estimated 25.6% in 2005–2006. Of note, older adults experienced the highest hospitalization rates (43.6%). Anticoagulants, with other two medication classes, were implicated in an estimated 46.9% of ED visits, which included clinically significant ADEs, such as haemorrhages. The authors reported that, since 2005–2006, the proportion of ED visits for ADEs from anticoagulants increased. Among older adults, three drug classes, including anticoagulants, were implicated in an estimated 59.9% of ED visits for ADEs. Furthermore, four anticoagulants (warfarin, rivaroxaban, dabigatran, and enoxaparin) were the most common drugs implicated in the ADEs.

A one-year retrospective chart review was conducted to determine the prevalence and severity of ADEs in patients presenting at EDs in two university-hospitals in the Canadian province of Newfoundland and Labrador (Sikdar et al., 2010). Of the 1,458 patients presenting to the EDs, 55 were determined to have an ADE. After a sample-weight adjustment, the prevalence of ADEs was found to be 2.4%. Prevalence increased with age (7.8%, ≥65 years) and the mean age for patients with ADEs was higher than for those with no ADEs (*p* < 0.01). A higher number of comorbidities and medications was associated with drug-related visits. CV agents (37.4%) were among the most common drug class associated with ADEs.

A cross-sectional study was performed in Canada to identify medications with a higher risk of ADEs among subjects aged ≥65 years, using public administrative data (Bayoumi et al., 2014). During the study period (2006–2008), among elderly patients in Ontario EDs, the NACRS (National Ambulatory Care Reporting System) identified more than 23,000 ADEs, which represented 0.8% of the sample (21.5% of them were hospitalised). Anticoagulants were among the drugs most frequently implicated in the ADEs of ED visits (14.2%).

In Asia, a prospective observational cohort study of patients aged 18 years and older presenting to the ED of an urban, tertiary medical center in Taiwan (Chen et al., 2012), was conducted to determine the incidence, risk and patient outcomes of ADE in an ED population. Of 58,569 ED visits, 452 patients (0.77%) had physician-documented ADE. CV agents accounted for the most ADE (25.8%) and consisted of 65.3% of ADE in patients aged 65 years and older. Elderly age resulted to be the main risk factor for ADE-related hospitalization (OR 1.9, 95% CI [1.1–3.4]).

Cheng and collaborators performed another prospective case-control study on elderly patients presenting to the ED in Taiwan (Chen et al., 2014). Out of 20,628 visits, physician documented a total of 295 ADEs in older adults. The number of administered drugs was identified as an independent risk factor for ADEs (OR 4.1, 95% CI [2.4–6.9] for 3–7 drugs; OR 6.4, 95% CI [3.7–11.0] for eight or more drugs). Moreover, diuretics, CV agents, and anticoagulants were the medications most commonly related to ADEs occurrence. In addition, a subsequent investigation revealed that the majority of older patients were males, reporting fatigue or altered mental status, with cardiovascular, renal, and respiratory complications, with a higher Charlson comorbidity index scores, and with a higher number of concomitant medications. Chen and colleagues reported that, among elderly, antithrombotic and CV agents were the drug groups most commonly associated ADEs (Chen et al., 2015).

In 2017, Oscanoa and co-workers performed a systematic review and meta-analysis of ADR-induced hospital admissions focusing on the elderly population (Oscanoa et al., 2017). They searched the literature from 1988 to 2015, identifying a total of 42 included articles, of which only 12 were conducted in the ED setting and none were focused on CV medications. Of note, the authors found that among the classes most frequently related to hospital admissions in the elderly were beta-blockers (1.8–66.7%), oral anticoagulants (3.3–55.6%), digoxin (1.6–18.8%), angiotensin-converting enzyme (ACE) inhibitors (5.5–23.4%), and calcium entry blockers (1.0–8.3%). Interestingly, as we observed in our sample, the majority of symptoms resulting in hospital admissions were: 1) hypotension, caused by beta-blockers, ACE inhibitors or calcium antagonists, 2) bleeding, due to oral anticoagulants utilization, or 3) bradycardia associated to the use of digoxin.

Considering the above comparison between our data and those published in other high-income countries, the relevant differences in standard of care and ED visit management policy need to be taken into account. In particular, differences in health care and ED payment system, ED crowding, and practices or plans to mitigate ED crowding must be considered (Pines et al., 2011). It has already been demonstrated that many patients living in high-income countries with good primary care and health insurance coverage, independently from the characteristics of each health care system, choose the ED over primary care, even for non-life-threatening conditions (Pines et al., 2011). Italy, such as many other high-income countries (i.e., Canada, Denmark, Finland, France, Saudi Arabia, Spain, Sweden, United Kingdom), presents a universal publicly funded health care system which is trying to prevent ED visits, and related crowding, for chronic conditions and adverse events associated to their pharmacological treatments.

Recently, a model for better understanding ADE-related ED visits was settled by Jatau and colleagues (Jatau et al., 2019). Authors identified a lack in knowledge and clinical practice, as well as targeted interventions to improve strategies for the prevention of ADEs. Their study underlined the need for a “proactive” role of healthcare professionals, in particular of clinical pharmacist expert in pharmacovigilance, to guarantee an optimal use of medications and to reduce the burden of ADEs as cause of ED visits. We believe that the active pharmacovigilance approach proposed in our study represents a first step toward Jatau’s suggestions, especially for elderly patients exposed to CV medications.

In summary, based on the evidence described in the present analysis and available in the scientific literature, Italian doctors should be aware that, among elderly outpatients exposed to CV medications, middle-old (75–84 years) and oldest-old (≥85 years) subjects, women, Caucasians, and subjects exposed to polypharmacy and suffering from one or more comorbidities represent the subgroups at higher risk of hospitalization. Considering the suspected drugs among CV medications, antiarrhythmics, beta-blocking agents, renin-angiotensin system inhibitors, antiplatelets, and anticoagulants are the classes most frequently involved in the ADE and associated to ED visits and hospitalization. Taking into consideration all these characteristics could be useful for general practitioners and specialists working in EDs to avoid and oversee CV medications-related ADEs in clinical practice involving the elderly.

## Strengths and Limitations

Like any retrospective analysis, this study also has some limitations. First, it contains only ADEs recognized and managed in ED. Second, since not all elderly patients experiencing an ADE, even if serious, go to the ED or spontaneously report the adverse event, an underestimation of ADEs could not be completely ruled out. This issue, is particularly relevant when we consider out-of-hospital mortality (i.e., home, nursing residence, etc.), especially sudden death which in the elderly can also be related to CV medications. Second, a selection bias of more clinically relevant cases (i.e., patients who referred to the ED after a contact with their general practitioner) could not be completely excluded. However, since we considered all serious and non-serious ADEs leading to the ED, the impact of this bias could be considered of relatively low relevance. Third, ADE reports may also be affected by inherent limitations, such as the quality of reported clinical data, which can sometimes be inaccurate or incomplete. Therefore, the absence of such data in the ADE reports may have impacted their clinical evaluation. For example, the lack of information regarding the level of consciousness (i.e., mental status) and eyesight, especially in the elderly where the risk of medication errors is higher as compared to younger population (i.e., medication with a narrow therapeutic window), could partially explain the risk of hospitalization observed in our sample. Moreover, since elderly patients suffering from CV diseases are certainly over represented in our sample, this evidence may not represent the entire elderly Italian population. However, in order to reduce this issue, comorbidities collected throughout the pharmacovigilance report forms were considered as covariates for adjustment in the multivariate logistic regression. Finally, we observed that the total number of participating centers was reached starting from 2011 (active monitoring at full capacity) (Supplementary Figures S1, S2). For this reason, during the first 4 years period, considering that we included only elderly patients treated with CV medications, we identified a small number of ADE reports. On the contrary, in the second (n = 8,762) and third (n = 8,124) 4 years period, the high number of ADE reports showed more homogeneous and representative results in terms of ED visits and risk of hospitalization. Nevertheless, since we aimed to perform an overall analysis of the data collected in ED throughout the active pharmacovigilance monitoring during the entire study period, we did not exclude, even if few, the data collected between 2007–2010.

Despite these limitations, this is the first analysis of its kind conducted in several Italian EDs and for a long period of time. The use of electronic ED medical records with high quality information on elderly population allowed us to adjust our analysis for important confounding variables, such as patients’ demographic characteristics, polytherapy, and comorbidities. In addition, the data we analyzed come from a large number of Italian EDs equally distributed throughout the five regions involved, which makes these evidences characteristic of and comparable to the whole elderly Italian population visiting the ED due to an ADE.

## Conclusion

Our real-world findings underline relevant safety aspects of CV medications in the elderly Italian population. ED clinicians must always consider the higher risk of hospitalization related to the use of CV drugs in elderly, particularly in oldest-old ones, for antiarrhythmics, beta-blocking agents, renin-angiotensin system inhibitors, antiplatelets, and anticoagulants.

Furthermore, our study confirms that the risk of hospitalization is significantly higher for all elderly age groups compared to young adults as the number of suspected and concomitant drugs, and the number of concomitant conditions increases.

Referring to the elderly, further analysis should be performed to evaluate the possible association between therapeutics guidelines changing over time and frequency/characteristics of ED visits and/or hospitalization due to ADEs.

In conclusion, we believe that in the elderly population there is still a need to increase the availability of evidence concerning potential ADEs due to inappropriate self-medication and ADEs due to drug-drug interactions and polytherapy. In our opinion, increasing the awareness of the risk of CV medications related ADEs is particularly important, especially for the general practitioner, who is frequently the first prescriber. In clinical practice, further active pharmacovigilance studies are needed to evaluate all safety aspects of drug use in the elderly.

## Data Availability

The datasets generated for this study are available on request to the corresponding author.

## MEREAFaPS STUDY GROUP

Members of the MEREAFaPS Study group who provided patient data for this study: Maria Luisa Aiezza, Alessandra Bettiol, Daria Bettoni, Corrado Blandizzi, Roberto Bonaiuti, Valentina Borsi, Annalisa Capuano, Errica Cecchi, Irma Convertino, Giada Crescioli, Martina Del Lungo, Cristina Di Mauro, Gabriella Farina, Sara Ferraro, Annamaria Fucile, Elena Galfrascoli, Elisabetta Geninatti, Linda Giovannetti, Luca Leonardi, Rosa Liccardo, Niccolò Lombardi, Anna Marra, Eleonora Marrazzo, Giovanna Monina, Alessandro Mugelli, Silvia Pagani, Maria Parrilli, Concetta Rafaniello, Francesco Rossi, Marco Rossi, Stefania Rostan, Marco Ruocco, Marita Sironi, Giulia Spada, Liberata Sportiello, Marco Tuccori, Alfredo Vannacci, Mauro Venegoni, Giuditta Violetta Vighi, Giuseppe Danilo Vighi.
