# Supplementary material for: Risk of Hospitalization Associated with Cardiovascular Medications in the Elderly Italian Population: A Nationwide Multicenter Study in Emergency Departments
Source: Front Pharmacol. 2021 Jan 29;11:611102. doi: 10.3389/fphar.2020.611102 (PMC7941274; doi:10.3389/fphar.2020.611102)
Supplement: Supplementary file 1 [file table1.docx]

**Supplementary table 1.** Characteristics of adult patients visiting the emergency department for an adverse drug event related to cardiovascular medications (ATC classes B and C).

| **Characteristics** | **Adults 18-64 years**  **N=3871 (%)** |
| --- | --- |
| **Sex** |  |
| Female | 1837 (47.5) |
| Male | 2034 (52.5) |
| **Patients’ Ethnicity** |  |
| Asian | 85 (2.2) |
| Black or African-American | 59 (1.5) |
| Caucasian | 3332 (86.1) |
| Others | 17 (0.4) |
| *Not available* | 378 (9.8) |
| **No. of suspected drugs involved in ADE** |  |
| 1 | 2931 (75.7) |
| 2 | 698 (18.0) |
| >3 | 242 (6.3) |
| **ATC class of suspected drugs** |  |
| ATC class B | 2062 (53.3) |
| ATC class C | 1749 (45.2) |
| Both classes | 60 (1.6) |
| **Concomitant drugs** |  |
| No | 1904 (49.2) |
| Yes | 1967 (50.8) |
| **No. of concomitant drugs** |  |
| 0 | 1904 (49.2) |
| 1 | 610 (15.8) |
| 2 | 421 (10.9) |
| 3-4 | 471 (12.2) |
| >5 | 465 (12.0) |
| **ATC class of most frequently reported concomitant drugs*** |  |
| *ATC class C* | *1305 (33.7)* |
| *ATC class A* | *768 (19.8)* |
| *ATC class N* | *449 (11.6)* |
| *ATC class B* | *470 (12.1)* |
| *ATC class M* | *179 (4.6)* |
| **Concomitant conditions** |  |
| No | 2482 (64.1) |
| Yes | 1389 (35.9) |
| **No. of concomitant conditions** |  |
| 0 | 2482 (64.1) |
| 1 | 682 (17.6) |
| 2 | 342 (8.8) |
| >3 | 365 (9.4) |
| **Most frequently reported concomitant conditions^#^** |  |
| *Arterial hypertension* | *520 (13.4)* |
| *Diabetes* | *155 (4.0)* |
| *Dyslipidaemia* | *149 (3.8)* |
| *Atrial fibrillation* | *103 (2.7)* |
| *Obesity* | *73 (1.9)* |
| *Chronic renal failure* | *67 (1.7)* |
| *Ischaemic cardiomyopathy* | *66 (1.7)* |
| **Presence of CAM** |  |
| No | 3831 (99.0) |
| Yes | 40 (1.0) |
| **Hospitalization** |  |
| No | 2837 (73.3) |
| Yes | 1034 (26.7) |

ADE: adverse drug events; ATC: Anatomical Therapeutic Chemical; CAM: complementary and alternative medicines.

*Most frequently reported concomitant drugs (as ATC class, 1^st^ level): A - alimentary tract and metabolism; B - blood and blood forming organs; C - cardiovascular system; M - musculo-skeletal system; N - nervous system.

^#^Most frequently reported concomitant conditions (as preferred terms) out of 2,815 reported low-level

terms MedDRA.
